# Cysteine-rich zinc finger proteins and the nuclear factor kappa-B pathway

**DOI:** 10.3389/fchbi.2024.1503390

**Published:** 2024-12-19

**Authors:** Andrew T. Stoltzfus, Sarah L. J. Michel

**Affiliations:** Department of Pharmaceutical Sciences, University of Maryland School of Pharmacy, Baltimore, MD, United States

**Keywords:** zinc finger proteins, inflammation, hydrogen sulfide, post-translational modification, persulfidation

## Abstract

Inflammation-related disorders, such as autoimmune diseases and cancer, impose a significant global health burden. Zinc finger proteins (ZFs) are ubiquitous metalloproteins which regulate inflammation and many biological signaling pathways related to growth, development, and immune function. Numerous ZFs are involved in the nuclear factor kappa-light-chain-enhancer of activated B cells (NFκB) pathway, associating them with inflammation-related diseases that feature chronically elevated pro-inflammatory cytokines. This review highlights the predominance of ZFs in NFκB-related signaling and summarizes the breadth of functions that these proteins perform. The cysteine-specific post-translational modification (PTM) of persulfidation is also discussed in the context of these cysteine-rich ZFs, including what is known from the few available reports on the functional implications of ZF persulfidation. Persulfidation, mediated by endogenously produced hydrogen sulfide (H_2_S), has a recently established role in signaling inflammation. This work will summarize the known connections between ZFs and persulfidation and has the potential to inform on the development of related therapies.

## 1 Introduction

**FIGURE 1:**
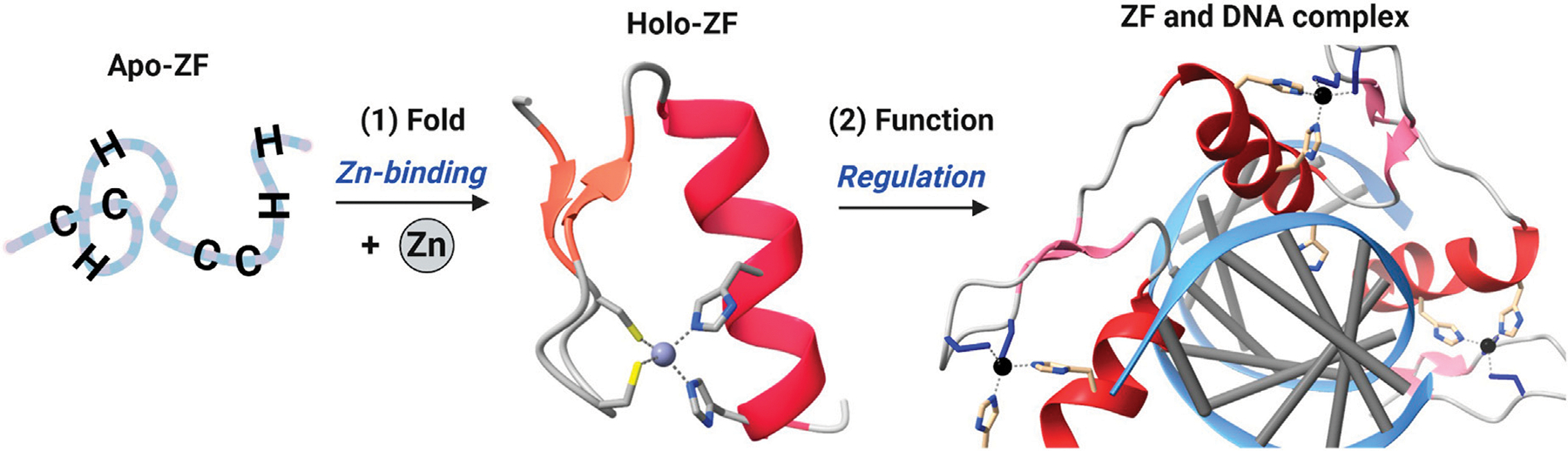
Cartoon diagram of zinc finger (ZF) domain structure and function. Zn(II) binding to a ZF domain results in a folded domain which can then recognize and bind to another macromolecule (often DNA or RNA) to function *via* regulation of transcription or translation. ZF shown is Zif268, PDB ID – 1AAY.

Metals are critically important to biological processes. In cells, metal-cofactored proteins or metalloproteins constitute about 30–40% of the total proteome, and new biological roles continue to be discovered (Rosato et al., 2016; Zhang and Zheng, 2020). Common metal co-factors in proteins include transition metals such as iron (Fe), zinc (Zn), copper (Cu), and alkali/alkali-earth metals such as sodium (Na), calcium (Ca) and potassium (K). Zn(II) is the second most common transition metal found in biology, after iron (Hierons et al., 2021; Jomova et al., 2022; O’Halloran and Culotta, 2000; Robinson and Glasfeld, 2020). Three roles have been ascribed to zinc: structural, catalytic and signaling. The structural role for zinc involves the metal binding to specific ligands of a protein leading to a defined protein structure. These proteins are called zinc finger (ZF) proteins (Lee and Michel, 2014) (Figure 1). The catalytic role involves zinc binding to specific ligands of a protein that includes an open site for substrate binding, with typical chemistry including hydrolysis and electron transfer; examples of these proteins include carbonic anhydrase, thermolysin and metallo β-lactamases (Coleman, 1998; Pettinati et al., 2016; Zastrow and Pecoraro, 2014). A signaling role for zinc has been invoked when describing zinc ‘sparks’ during embryonic development and, more broadly, for cell cycle progression (Lo et al., 2020; Que et al., 2015; Warowicka et al., 2022; Yang et al., 2013; Zinatizadeh et al., 2021). Similarly, ZFs contribute to multiple signaling pathways by functioning as transcriptional, translational, and post-translational regulators. ZF dysfunction has been linked to numerous pathologies, including developmental, neurodegenerative, and immune disorders (Behrens and Heissmeyer, 2022; Bu et al., 2021; Cassandri et al., 2017; Patial and Blackshear, 2016; Sun, 2017; Todorova et al., 2015; Vilas et al., 2018; Yoshinaga and Takeuchi, 2019). These conditions are associated with chronic inflammation and increased inflammatory cytokines (TNFα, interleukins, etc.), in part from persistent activation of the NFκB signaling pathway.

The NFκB pathway is stimulated by a plethora of endogenous and exogenous agonists (i.e., TNFα and LPS, respectively) which ultimately lead to formation of an NFκB complex that translocates from the cytoplasm to the nucleus to act as a transcriptional activator of stress-response genes. ZFs are heavily involved in both the transduction (pro-inflammatory) and the repression (anti-inflammatory) of the signal. Post-translational modifications (PTMs), such as phosphorylation and ubiquitination, are essential to NFκB signaling and mediated, in part, by ZF activity. As ZFs are Cys-rich domains, they are also subject to several sulfur centered modifications, such as nitrosylation (Cys-SNO), persulfidation (Cys-SSH), and glutathionylation (Cys-SSG) (Checconi et al., 2019; Dyer et al., 2019; Vignane and Filipovic, 2023). There are many ways for the cell to adjust ZF function *via* PTMs and modulate their regulation of signaling. This review discusses the role of ZFs throughout the NFκB pathway, their associations with disease, and the functional implications of ZF persulfidation, a recently identified PTM of ZFs (Li et al., 2024; Stoltzfus et al., 2024).

## 2 Zinc finger proteins: origins, classifications, and ubiquity in mammalian biology

**FIGURE 2:**
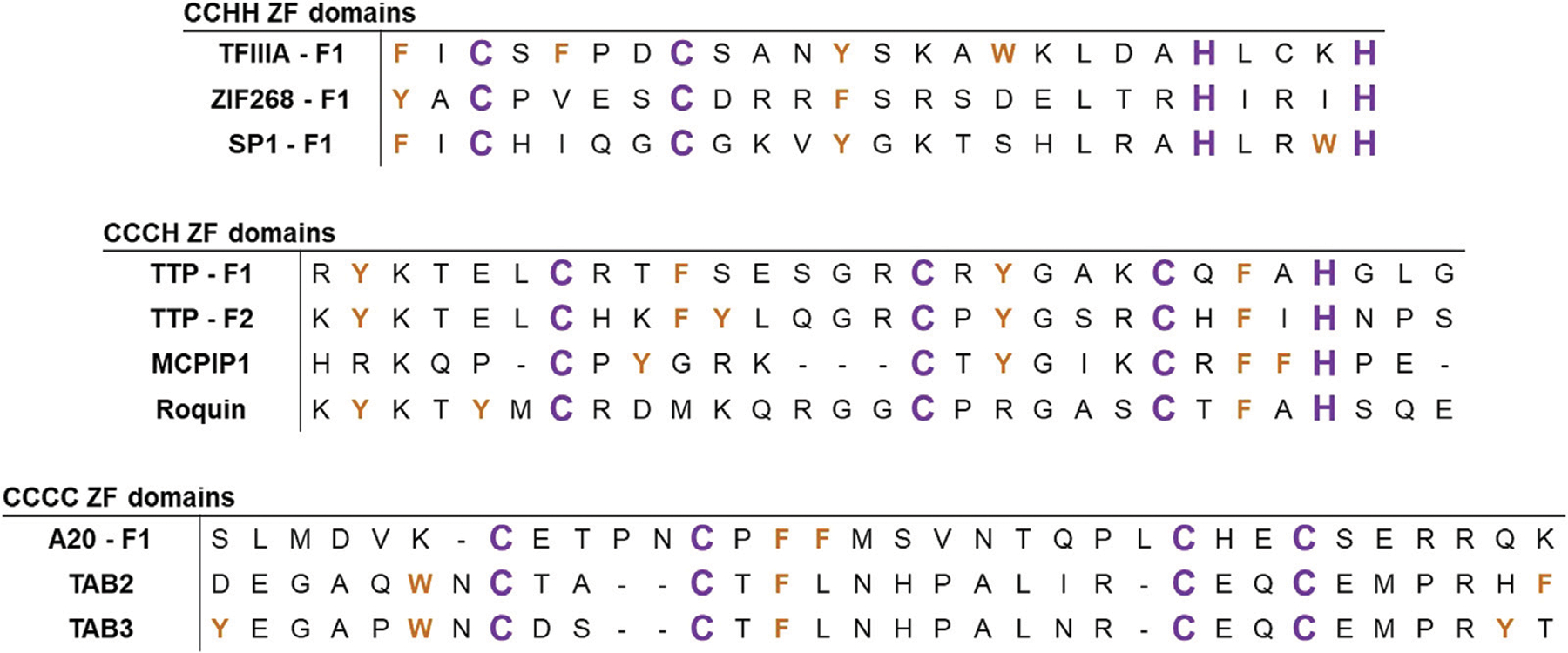
Alignments of the ZF sequences of several representative ZFs. Sequences are aligned based on C/H domains, with examples of ZFs involved in NFκB signaling. Zn(II)-coordinating ligands (C and H) are colored in purple and aromatic residues (W, Y, and F) are colored in orange.

ZF proteins contain conserved repeats of four cysteine (Cys, C) and/or histidine (His, H) residues within their primary amino acid sequence (Besold et al., 2010; Michalek et al., 2011). These residues serve as ligands to coordinate zinc in a tetrahedral geometry resulting in a folded protein (Lee and Michel, 2014). The first protein to be identified as a ZF was transcription factor IIIA (TFIIIA) from *Xenopus laevis* (Miller et al., 1985; Taylor and Segall, 1985). TFIIIA contains eight ZF domains with a sequence of CX_4_-CX_12_-HX_3_-H along with a singular domain of CX_4_-CX_6_-HX_5_-H (Shastry, 1996). Bioinformatic comparisons of TFIIIA’s sequence led to the structural proposal of finger-shaped domain repeats which could bind non-coding regions of DNA and regulate downstream transcription (Berg, 1986). The first ZF crystal structure of Zif268, in addition to subsequent structures of TFIIIA and related homologs, established the ββα fold of the “classical” ZF domain (Foster et al., 1997; Pavletich and Pabo, 1991). Upon zinc binding, the classical ZF adopts a structure in which each domain has a ββα fold with a hydrophobic core made up of three conserved aromatic residues (W, Y, F) preceding the first Cys and in the CX_12_-H linker region (Figure 2, *top*) (Padjasek et al., 2020). Classical (CCHH) ZFs are transcription factors, and each ZF domain binds to specific GC-rich DNA sequences *via* hydrogen bonding interactions between side chains on the α helices and bases on the DNA (Chabert et al., 2019; Kluska et al., 2018; Negi et al., 2023).

**FIGURE 3:**
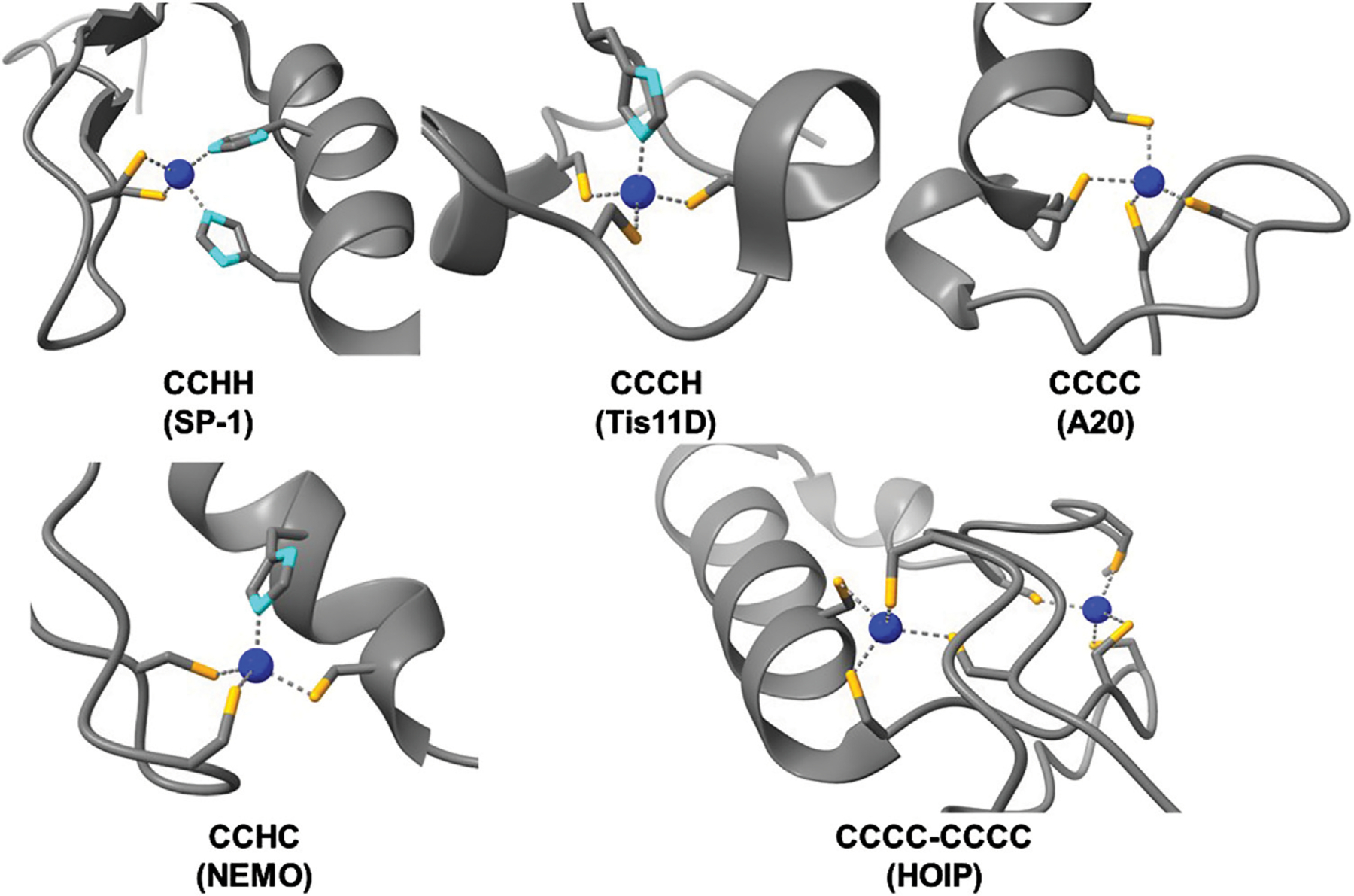
Structures of zinc bound ZF domains of varying Cys/His compositions (Cys-sulfur atoms are colored in orange, His-nitrogen atoms are colored in light blue, and Zn(II) atoms are colored in dark blue). PDBs used, from top left to bottom right: SP1 – 1VA1, Tis11D – 1RGO, A20 – 3OJ3, NEMO – 5AAY, HOIP – 6SC6.

Since the discovery of CCHH ZFs in the 1980s, more than 30 types of non-classical ZFs have been identified. These ZFs are predominantly known for RNA binding and other forms of post-transcriptional regulation. Non-classical ZFs contain different combinations of Cys and His ligands (e.g., CCCH, CCHC, and CCCC) with varied spacing between the ligands (Michalek et al., 2011; Padjasek et al., 2020; Pritts and Michel, 2022) (Figure 2, *middle and bottom)*. In total, ZF domains exhibit a diverse array of peptide folds and DNA/RNA binding modalities across the multitude of classes and structures (Figure 3) (Lee and Michel, 2014; Ok et al., 2021; Yoshinaga and Takeuchi, 2019). As such, ZFs are integral regulators of cell signaling at the level of transcription, translation, and post-translation (i.e., stabilizing and/or destabilizing ubiquitination) (Fennell et al., 2018; Kaczynski et al., 2003; Louis et al., 2021; Pikkarainen et al., 2004; Tu et al., 2019; Yang et al., 2013).

As the complexity of organisms increases, so too does the abundance of ZFs within the respective proteomes (Andreini et al., 2006b). This abundance and diversity of ZFs is exemplified in humans, where 8.9% of all proteins contain at least one ZF domain (Andreini et al., 2006a; Bertini et al., 2010). The abundance of Cys residues relative to all amino acids similarly increases with organism complexity, with humans having the highest proportion, at 2.3% Cys (Garrido Ruiz et al., 2022; Go et al., 2015; Wiedemann et al., 2020). This increase in ZFs may be related to the increase in Cys sites, as Zn(II) is redox inert, offering protection for redox sensitive Cys thiols during oxidative stress. The increased frequency and utilization of ZF domains highlights their importance in biological development and cell signaling pathways.

**FIGURE 4:**
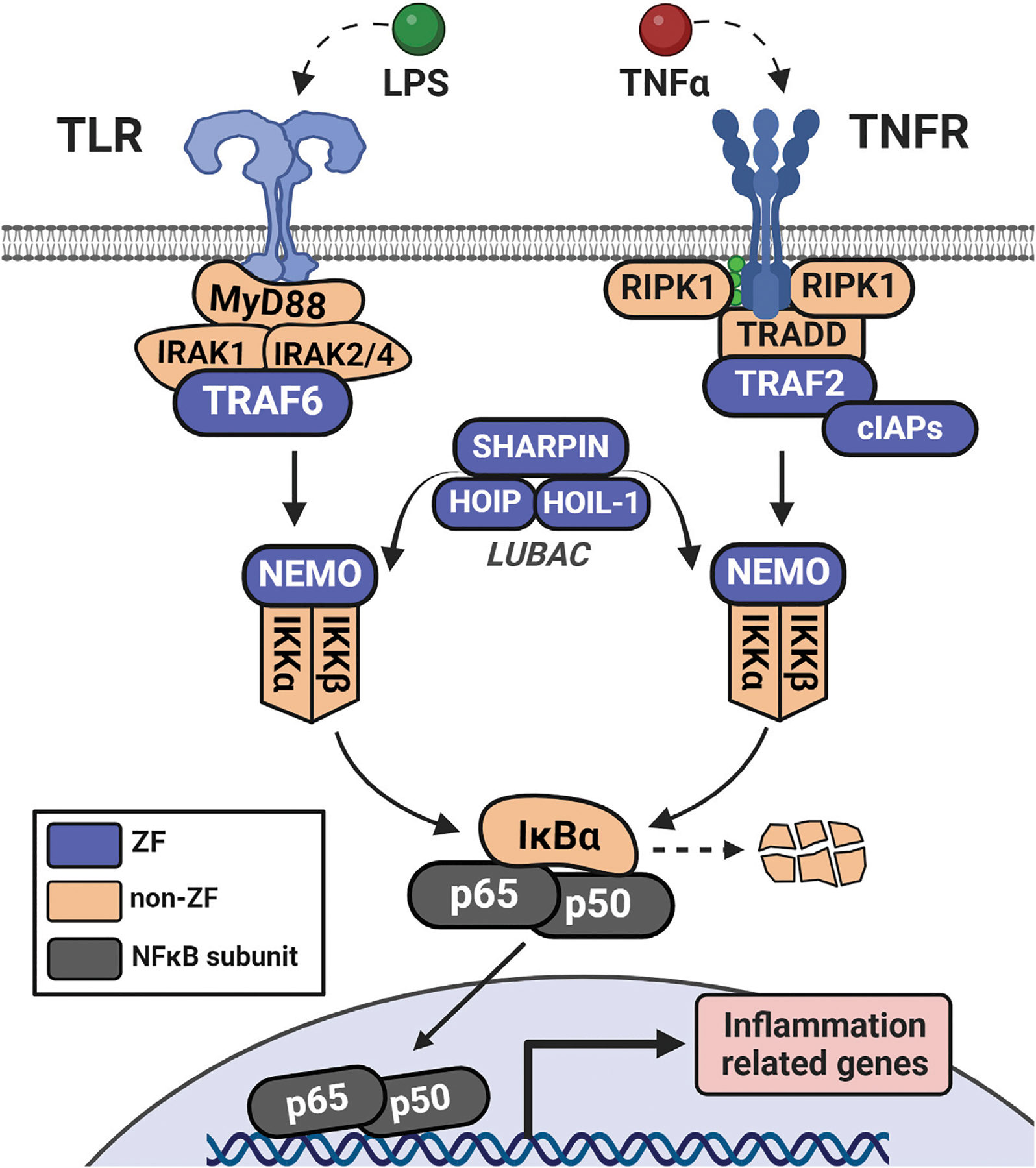
Initiation of NFκB signaling *via* LPS or TNFα involves distinct receptor-adaptor complexes that both lead to activation of the IKK complex (IKKα, IKKβ, and NEMO). This complex phosphorylates IκBα which leads to its ubiquitination and degradation, de-repressing the NFκB complex (in this case, p50 and p65) and allowing it to translocate to the nucleus. NFκB then acts as a transcription factor to upregulate a host of inflammation-related genes.

One pathway for which ZFs play key roles is the nuclear factor-kappa B (NFκB) signaling pathway, where ZFs modulate NFκB activity throughout the phases of stimulation, signal transduction, and resolution (Figure 4) (Tokunaga et al., 2011). ZFs are associated with immune modulation, apoptosis, cell proliferation, and cancer (Guo et al., 2017; Jarosz et al., 2017; Kang et al., 2021; Lipkowitz and Weissman, 2011; Park et al., 2018; Zhao et al., 2019). There are a variety of stimuli for distinct NFκB subunits, leading to canonical or non-canonical NFκB activation. Canonical activation occurs when the NFκB subunits p65 and p50 are liberated from their NFκB-inhibitor-alpha (IκBα)-mediated cytoplasmic restriction, translocate to the nucleus, and dimerize to act as a transcriptional regulator (Lu et al., 2021). The non-canonical pathway initiates with signal-induced p100 processing, regulated by NFκB-inducible kinase (NIK)-dependent phosphorylation, leading to non-canonical NFκB subunits p52 and RELB translocating and upregulating transcription (Sun, 2017). There is overlap of the stimuli and receptors (for example, TNFα/TNF-receptor family) which lead to activation of either pathway, and thus NFκB signaling often occurs *via* both mechanisms simultaneously rather than discretely (Lu et al., 2021; Tao et al., 2022). This review focuses on the canonical NFκB pathway and associated ZFs.

## 3 ZF regulation of the NFκB signaling pathway

### 3.1 Human proteome sorting of ZFs involved in NFκB signaling

To identify the ZFs that play roles in NFκB signaling, the full list of reviewed *H. sapiens* proteins in Uniprot (n = 20,428 total proteins) was filtered based upon the presence of at least one annotated ZF domain (n = 1,814 total proteins with ZF annotation). This list of ZFs was then analyzed using DAVID software (NIH), a web-based server for functional enrichment analysis of gene lists, using *H. sapiens* as the background gene list to match the Uniprot accessions (Sherman et al., 2022). With the gene list in DAVID, pathway analysis by the Kyoto Encyclopedia of Genes and Genomes (KEGG) knowledge database was selected (Kanehisa and Goto, 2000). KEGG analysis was employed because it yielded the highest number of gene hits (17) for ZFs in the NF-kappa B signaling pathway (KEGG designation hsa04064; Table 1; Supplementary Table S1).

Of the 17 associated gene hits, 13 are pro-inflammatory in their NFκB function, indicating that the KEGG pathway analysis is limited in its included proteins to early signaling/pathway activation. There are additional NFκB-responsive ZFs that were not identified in the KEGG analysis which are included in Table 1 and are discussed in this review. These proteins include Tristetraprolin (TTP), MCP-1-induced protein-1 (MCPIP1), and Roquin, which negatively regulate the pro-inflammatory cytokines and chemokines produced by NFκB activation (Fu and Blackshear, 2017; Makita et al., 2021; Xu et al., 2012). Ubiquitin-editing ZFs are also included, such as the anti-inflammatory mediators optineurin (OPTN) and OTU domain-containing protein 7A (OTU7A), as well as the pro-inflammatory ZFs SHARPIN, HOIL-1L, and HOIP. The latter three form the linear ubiquitination chain assembly complex (LUBAC) (Fennell et al., 2018; Tokunaga et al., 2011) which catalyzes many forms of protein ubiquitination, enabling protein-protein interactions (PPIs) required for NFκB activation. M1-linked linear polyubiquitination (LUBAC) and K63-linked ubiquitination (LUBAC and others) are forms of ubiquitination that are unique to canonical NFκB activation (Sun, 2017; Tao et al., 2022). Eight other ZFs in the list from Table 1 have ubiquitin E3 ligase activity, demonstrating the essentiality of this PTM, and ZFs, in NFκB signaling.

All of the ZF gene hits from the DAVID analysis, along with the additional ZFs described above, are included in this holistic review that focuses on the importance of ZFs in NFκB signaling and related pathways (i.e., apoptosis, cancer, etc.).

### 3.2 Activation of pathway signaling

**FIGURE 5:**
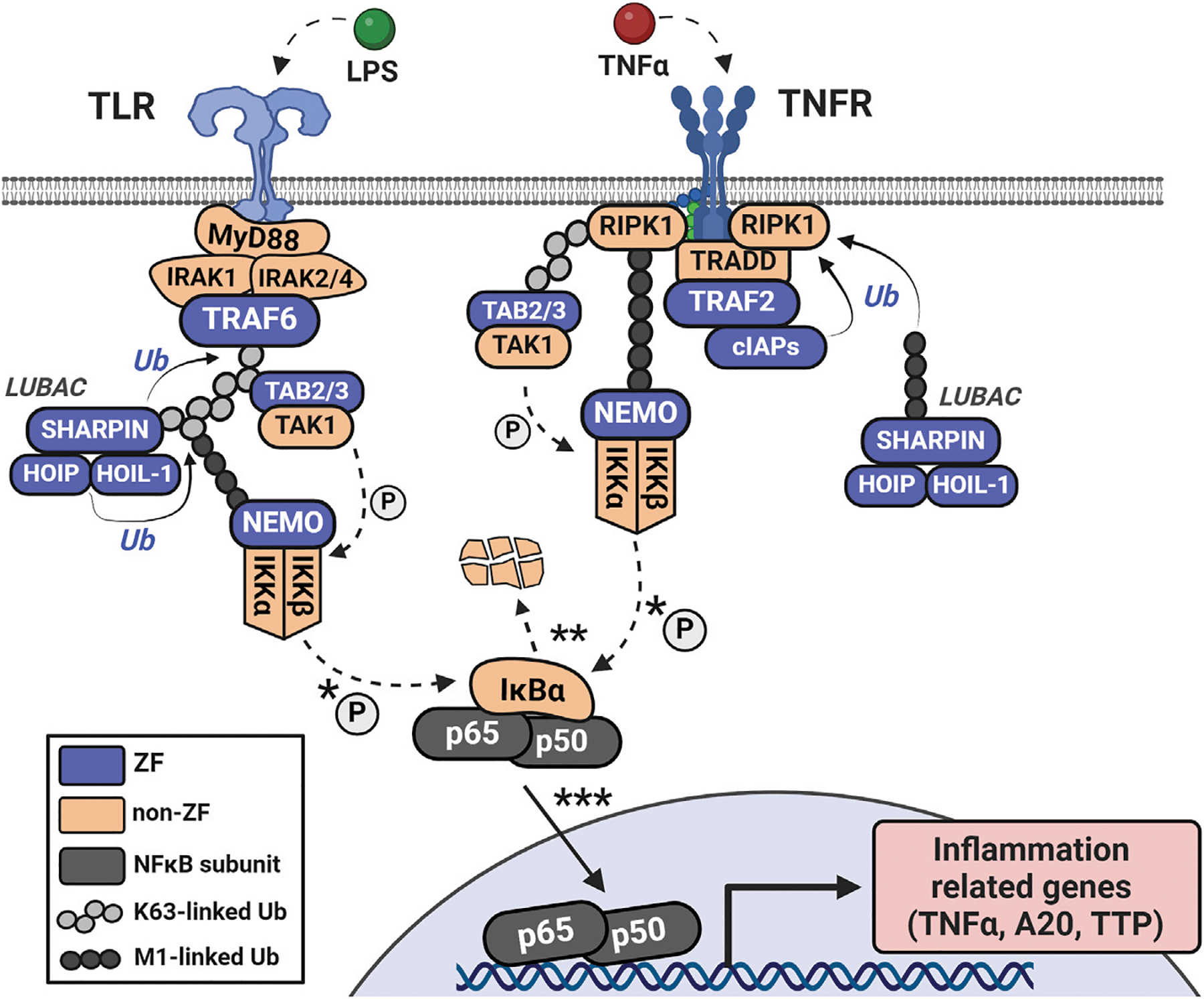
Upon TLR activation, the MyD88 adaptor complex forms with IRAK1/2 and TRAF6. In TNF-mediated NFκB signaling, TNFR recruits an adaptor complex of TRADD, TRAF2, RIP1, and cellular inhibitors of apoptosis (cIAP1 and cIAP2). The IAP ZFs have K63-ubiquitin ligase activity and modify RIP1. Ubiquitination of RIP1 or TRAF6 auto-ubiquitination signals recognition by the RanBP2 ZFs of TAB2/3 which specifically recognize K63-linked ubiquitin. The K63-linkages create a scaffold for interaction with the LUBAC complex (SHARPIN, HOIP, and HOIL-1 each having Ubiquitin-associating domains) and recognition by the N-terminal CCHC ZF domain of NEMO. M1-linked ubiquitination via LUBAC supplements K63-linked ubiquitination, functioning similarly to stabilize substrates. The interaction of NEMO with these ubiquitinated elements activates the IKK complex, which phosphorylates and degrades IκBα, leading to the nuclear translocation of the p50/p65 NFκB complex.

The tumor necrosis factor receptor (TNFR)-associated factors (TRAFs) are a family of ZFs that act as early transducers of NFκB signaling. All members contain multiple CCCC ZF domains and one RING finger domain which connects various stimulated receptors (for example, TNFRs and toll-like receptors, TLRs) with their respective adaptor complexes (TRADD and MyD88, respectively) (Shi and Sun, 2018). In TLR initiation, activated MyD88 forms a complex with and activates IRAK1/2, which is recognized by TRAF6 (Figure 5). The RING finger domains of TRAF6 function as E3 ligases, in tandem with the E2 complex Uev1A:Ubc13, to catalyze the K63-linked ubiquitination of TRAF6 and other substrates (Xie, 2013). K63-linked TRAF6 is recognized by the ZF complex of TGF-beta activated kinase 1 (TAK1) through its subunits of TAK1-binding proteins 2 and 3 (TAB2/3), both containing a single RanBP2 (CCCC-type) ZF which recognizes K63-linked ubiquitin (Park, 2018). This complex is then recognized by NFκB-essential modulator (NEMO) through NEMO’s lone CCHC ZF domain on the N-terminus (Maubach et al., 2017). Interaction of NEMO with the ubiquitinated upstream elements activates the IκBα kinase complex (IKK), which leads to phosphorylation and degradation of IκBα by IKK, and nuclear translocation of the p50/p65 NFκB complex.

For TNF-mediated activation of NFκB signaling, TNFR forms a signaling complex with TNF receptor-associated death domain (TRADD), TRAF2 or TRAF5, receptor-interacting protein kinase (RIP1), and the cellular inhibitors of apoptosis (cIAP1 and cIAP2) (Shi and Sun, 2018) (Figure 5). The IAP family are ZFs with E3 ubiquitin ligase activity which ubiquitinate RIP1 and recruit LUBAC (Gyrd-Hansen and Meier, 2010). LUBAC is comprised of 3 ZFs: SHANK-associated RH domain-interacting protein (SHARPIN), heme-oxidized iron regulatory protein ubiquitin ligase (HOIL-1), and HOIL-1 interacting protein (HOIP) (Fuseya and Iwai, 2021; Kasirer-Friede et al., 2019). Both HOIL-1 and HOIP have an arrangement of the following ZF domains: RING1, In-Between-RING (IBR), and RING2; these domains confer the E3 ubiquitin ligase functionality. LUBAC’s subunits also have domains that promote and stabilize their trimeric association, such as the ubiquitin-associating domain of HOIP and the ubiquitin-like domains of HOIL-1 and SHARPIN. LUBAC can uniquely conjugate linear ubiquitin to substrates via the Met1 residue of free ubiquitin; this serves as an additional scaffold for NEMO and others to recognize which is distinct from the branched chains of K63-ubiquitination (Fuseya and Iwai, 2021; Tokunaga et al., 2011).

In both TNFR-mediated or TLR-mediated activation, ubiquitin serves as both a scaffold to relay the signal from receptor to effector (NFκB complex) and to catalyze the degradation of the NFκB inhibitor (IκBα). IKK-mediated degradation of IκBα, and subsequent liberation of p50 and p65 (canonical NFκB subunits) for transcriptional activity, occurs within minutes, leading to a rapid transcriptional activation of NFκB-associated genes (Beg and Baldwin, 1993; Chen et al., 1995). Elevated levels of cytokine mRNAs (e.g., TNFα, interleukins, etc.) are efficiently translated into proteins, acting as positive feedback of this pathway, as RNA-regulatory ZFs, such as tristetraprolin (TTP), are not yet fully responsive (Rappl et al., 2021). Expressed cytokines and chemokines can also participate in crosstalk with adjacent cells, tissues, etc. This pro-inflammatotory signaling is required to activate the immune system and/or combat invading pathogens.

### 3.3 Regulation of pro-inflammatory stimuli

**FIGURE 6:**
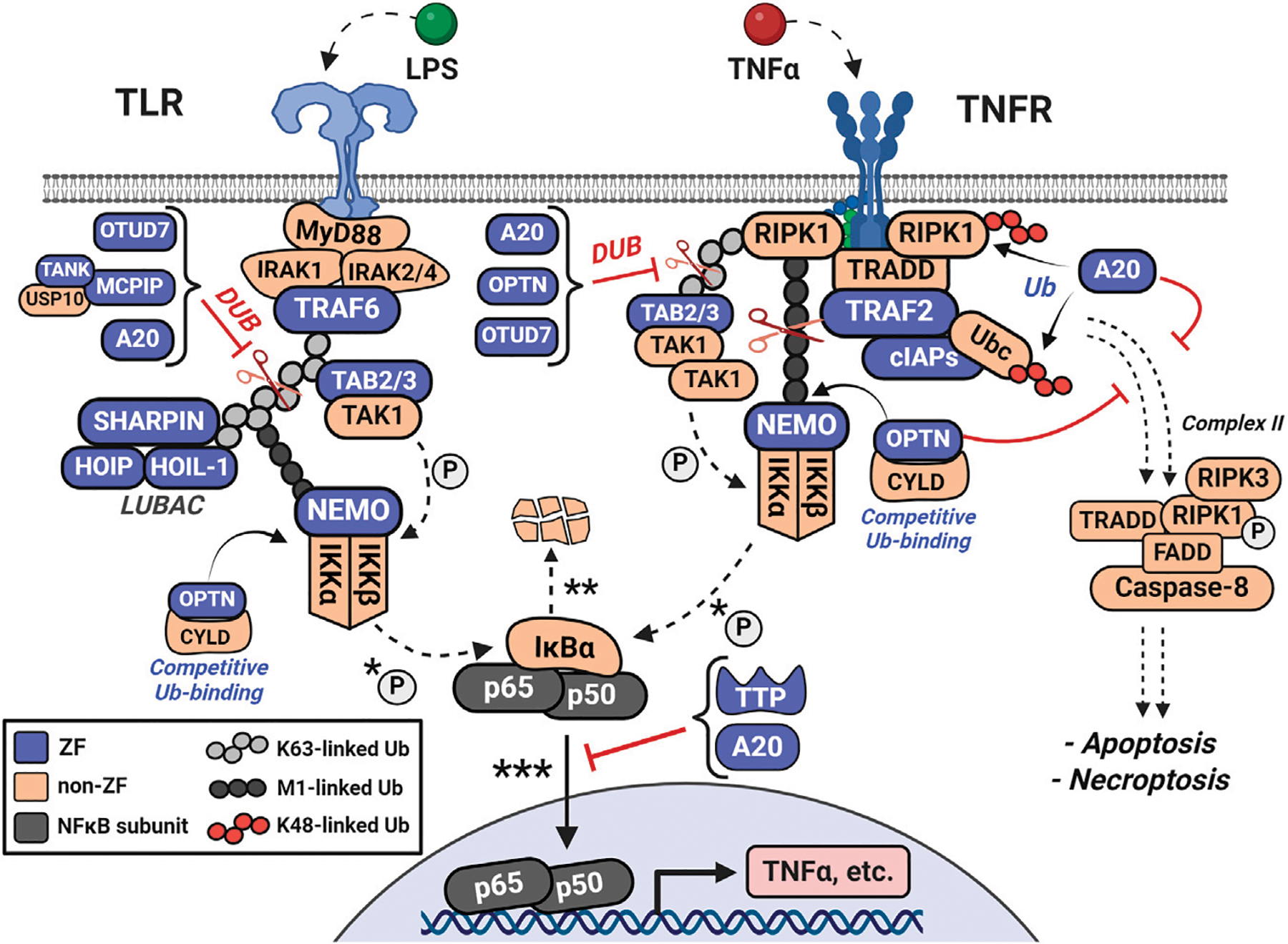
Ubiquitin-editing proteins disrupt NFκB signaling by inhibiting pro-inflammatory interactions among ubiquitinated proteins and by degrading pro-inflammatory mediators *via* K48-ubiquitination. A20 is rapidly upregulated and acts as both a deubiquitinase (DUB) for M1 and K63-ubiquitin linkages (i.e., of LUBAC and NEMO) and as an E3 ligase for K48-ubiquitin linkages (i.e., RIPK1 and Ubc). Additional ubiquitin-associated proteins include Optineurin (OPTN), OTUD7, and Cylindromatosis (CYLD). OPTN shares high sequence homology with NEMO and competes for M1-and K63-linked polyubiquitin. CYLD, OTUD7, and MCPIP also contribute to ubiquitin-related regulation of the pathway. Together, these ubiquitin-editing enzymes counteract the actions of the cIAP and TRAF families, LUBAC, and NEMO in regulating ubiquitination.

It is important that levels of pro-inflammatory stimuli are controlled by repressors of the pathway, which are also induced by NFκB, to dampen signaling. As shown in Figure 6, one subset of anti-inflammatory mediators are the ubiquitin-editing proteins, including TNFα-induced protein 3 (TNFAIP-3, A20). A20 is a CCCC-type ZF which is rapidly upregulated to act as both a deubiquitinase (DUB) of M1 and K63-ubiquitin linkages and an E3 ligase of K48-ubiquitin linkages (Mooney and Sahingur, 2021; Priem et al., 2020). A20 recognizes many forms of ubiquitinated proteins and disrupts multiple pathway transducers by inhibiting their interactions through ubiquitin. Additionally, it can conjugate K48-ubiquitin chains to early pathway inducers, degrading the substrate, further disrupting the pro-inflammatory signal (Verstrepen et al., 2010). A20 has seven CCCC-type ZF domains, where ZF 4 confers the K48-linked ubiquitin ligase activity and ZF 7 is crucial for polyubiquitin recognition and deubiquitination (Priem et al., 2020). A20 quickly functions to disassemble the ubiquitin frameworks that are the structural basis for continued signaling.

Additional ubiquitin-associated proteins are the ZFs Optineurin (OPTN) and OTUD7, as well as the non-ZFs Cylindromatosis (CYLD), and OTULIN (Fennell et al., 2018). OPTN’s C-terminal UBAN and CCHC ZF domains are required for interaction with ubiquitin-like structures, including linear polyubiquitin and CYLD (Guo et al., 2020). OPTN and NEMO have high sequence homology and signal the NFκB pathway antagonistically through their competition for both linear and K63-linked polyubiquitin (Qiu et al., 2022; Slowicka and van Loo, 2018). CYLD and OTULIN negatively regulate NFκB activity through recognition and deubiquitination of polyubiquitinated substrates, including the TRAFs and HOIP (Fennell et al., 2018; Shi and Sun, 2018; Xie, 2013). Collectively, these ubiquitin-editing enzymes regulate ubiquitination antagonistically to NEMO, the LUBAC complex, and the TRAF family.

**FIGURE 7:**
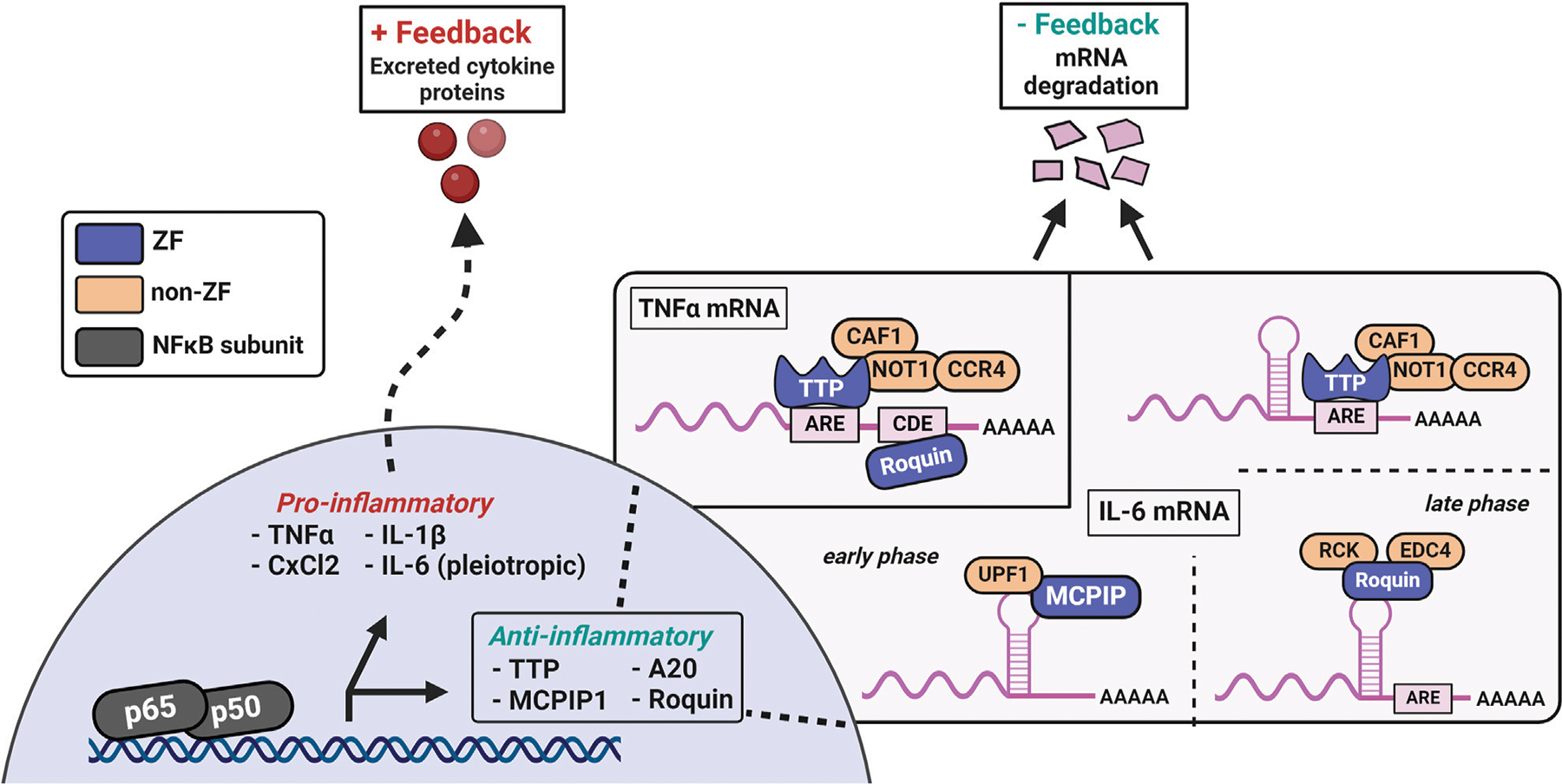
TTP, MCPIP, and Roquin are induced by NFkB transcriptional activity and negatively regulate excessive pro-inflammatory mediators. MCPIP1 has a single CCCH ZF domain for mRNA recognition, paired with an adjacent PIN RNase domain for mRNA degradation. TTP features two tandem CCCH ZFs that are crucial for recognizing AU-rich sequences in the 3′-UTR of mRNAs. TTP’s ZFs are also necessary for localization to the nucleus and mRNA-processing bodies. The degradation of cytokine mRNAs by TTP involves a conserved C-terminal domain that recruits the CCR4-NOT deadenylase complex to the TTP-bound mRNA. Additional modes of TNFα and IL-6 mRNAs regulation are evident in Roquin, which recognizes conserved stem-loop structures and the constitutive decay element (CDE) in cytokine mRNAs. Roquin’s binding recruits the EDC4 and RCK proteins for deadenylation, an event which destabilizes mRNAs and leads to their degradation in the cases of regulation by TTP and Roquin.

While the DUBs act to prevent further NFκB translocation and activation, RNA-binding ZFs are induced to respond to the NFκB-dependent mRNAs (Behrens and Heissmeyer, 2022; Maeda and Akira, 2017) (Figure 7). TTP and others regulate the positive feedback of cytokine mRNAs by limiting their translation into mature proteins which are capable of further stimulating NFκB-related receptors (ex: TNFα and various interleukins). These RNA-binding ZFs recognize and bind specific ribonucleotide sequences, destabilizing the mRNAs for translation, and ultimately leading to their degradation (Hudson et al., 2004; Kedar et al., 2012; Lai et al., 2000). The complete resolution of these cytokines to basal levels can take upwards of 4 h, depending on the initial concentration and identity of the stimuli (e.g., LPS, TNFα, etc.) (Mulvey et al., 2021).

TTP, MCPIP, and Roquin are CCCH-type ZFs which negatively regulate excessive pro-inflammatory mRNAs and are essential for proper immune function (Maeda and Akira, 2017; Makita et al., 2021; Rappl et al., 2021). TTP contains two CCCH ZF domains which are required for specific recognition of AU-rich sequences in the 3′-UTR of mRNAs (Hudson et al., 2004). TTP’s ZFs are also necessary for localization to the nucleus and mRNA-processing bodies (Lai et al., 2000). Degradation of cytokine mRNAs requires an evolutionarily conserved C-terminal domain which recruits the CCR4-NOT deadenylase complex to TTP-bound mRNA (Fabian et al., 2013). In addition to mRNA destabilization, TTP disrupts NFκB nuclear translocation and participates in alternative splicing in innate immunity (Gu et al., 2013; Schichl et al., 2009; Tu et al., 2020). In canonical NFκB signaling, TTP is one of the main anti-inflammatory mediators due to its simultaneous regulation of thousands of ARE-containing mRNAs (Brooks and Blackshear, 2013; Rappl et al., 2021; Tiedje et al., 2016).

MCPIP1, has an N-terminal ubiquitin-associated domain, a single CCCH ZF domain for mRNA recognition, and an adjacent PIN-like RNase domain for mRNA degradation (Fu and Blackshear, 2017). MCPIP can regulate the NFκB pathway by deubiquitination of TRAF complexes, *via* its ubiquitin-associated domain, and by degrading cytokine mRNAs, with its inherent endonuclease activity from the adjacent ZF-RNase domains (Xu et al., 2012). Roquin contains two different ZFs: one CCCH domain adjacent to a ROQ domain and one RING ZF domain. The CCCH ZF and ROQ domains are required for mRNA target recognition and recruitment of the decapping complex of mRNA-decapping protein 4 (EDC4) and RCK. The RING ZF is proposed to be important for E3-ubiquitin ligase activity and stress granule association (Zhang et al., 2015). Roquin recognizes conserved stem-loop elements in cytokine mRNAs, such as TNFα, which are spatially distinct from the ARE’s regulated by TTP and represent an overlap in regulation (Maeda and Akira, 2017). MCPIP1 and Roquin also interact in a way that affects the autoregulation of their own mRNAs (Behrens et al., 2021). In addition to the autoregulation of *mcpip1* mRNA by MCPIP1, *ttp* mRNA has multiple ARE elements in its 3′-UTR, which serve as regulatory sites for TTP-mediated degradation. Collectively, these CCCH ZFs are induced by NFκB activation and disrupt the positive feedback loop through degradation of cytokine mRNAs. At some point in the resolution process, activity of these RNA-binding ZFs decreases, and downregulation of their levels restores pathway homeostasis.

## 4 Post-translational modifications of ZFs in NFκB signaling

### 4.1 Phosphorylation

ZFs are signaled *via* PTMs of the ZF domain, or adjacent motifs in the full-length sequence, which affects overall protein function and cell signaling (Fennell et al., 2018; Vignane and Filipovic, 2023; Vilas et al., 2018). In the NFκB pathway, phosphorylation of Ser or Thr residues signals through several ZFs, fine-tuning their activity for a regulated inflammatory response. ZF kinases such as the TAB family (TAB, subunits of TAK1) and protein kinase C family (PKC), in addition to the Ca(II)-mobilizing ZF bruton tyrosine kinase (BTK), phosphorylate key substrate proteins to facilitate the early NFκB signal (Altman and Kong, 2014; Kramer et al., 2023; Newton, 2018). For example, the upstream kinases phosphorylate and activate the IKK complex, which then phosphorylates IκBα, leading to ubiquitination and degradation of IκBα, liberating NFκB to translocate to the nucleus (Beg et al., 1993; Chen et al., 1995). Phosphorylation of TTP by MAPK kinases (e.g., MK-2) at S60 and S186 inhibits TTP function *via* recruitment of the 14-3-3 adaptor complex, permitting the transient increase of cytokine mRNAs during the early inflammatory response (Sandler and Stoecklin, 2008; Tiedje et al., 2014). TTP is then dephosphorylated by PP2A, which directly competes with the 14-3-3 complex, releasing TTP for mRNA regulation.

### 4.2 Ubiquitination

Another common PTM communicated through ZFs in the NFκB pathway is ubiquitination, which occurs in at least three distinct forms, namely, K48, K63, and M1-linked ubiquitination (Maubach et al., 2017). K48-linked ubiquitination of proteins signals them for degradation *via* the proteasome. This PTM of IκBα is required to de-repress NFκB and allow for nuclear translocation (Beg and Baldwin, 1993; Beg et al., 1993; Chen et al., 1995). K48-linked ubiquitination is also used by A20, the IAPs, and the TRAFs to regulate early signaling complexes. In contrast, K63-linked ubiquitin and the linearly linked M1-ubiquitin stabilize their substrates and signal as structural scaffolding for downstream interactions of co-activators (i.e., NEMO and SHARPIN) or co-repressors (i.e., TRAF6 and A20) (Fuseya and Iwai, 2021; Kasirer-Friede et al., 2019). In total, the variety of ubiquitination is intertwined with ZF regulation of the NFκB signaling, particularly the RING ZF E3 ligase domains.

### 4.3 Redox-associated PTMs, cysteine abundance, and persulfidation

Much like phosphorylation and ubiquitination, cysteine-specific PTMs play a role in modifying protein activity during inflammatory signaling, particularly for cysteine-rich ZFs (Chantzoura et al., 2010; Kukulage et al., 2022; Oppong et al., 2023). Human proteins have the highest abundance of cysteines relative to other life forms, and ZF motifs are particularly enriched in their cysteine content (Wiedemann et al., 2020) (See Table 2; Supplementary Table S2). Cysteine residues can be oxidized to disulfides, and while Zn(II) binding to these cysteine residues decreases the susceptibility of cysteine oxidation, such redox changes can occur affecting ZF integrity and function during stress (Doka et al., 2020; Hartle et al., 2016; Kimura, 2015; Kumar and Banerjee, 2021). Adequate redox balance through cellular glutathione and other redox mediators is essential for mitigating oxidative damage to ZFs and other metalloproteins (Fukuto et al., 2020). One cysteine-specific PTM is persulfidation (Cys-SH to Cys-SSH), which modulates protein function and protects the cysteine thiols from oxidation during oxidative stress (Chantzoura et al., 2010; Luo et al., 2023).

Persulfidation is mediated through the gaseous signaling molecule, hydrogen sulfide (H_2_S). Cystathionine-gamma-lyase (CSE), cystathionine-beta-synthase (CBS), and 3-mercaptopyruvate sulfurtransferase (3-MST) are the main H_2_S synthase enzymes. These enzymes constitute the transsulfuration pathway governing the homeostasis of H_2_S and related sulfur-containing small molecules (Kimura, 2015; Vignane and Filipovic, 2023). H_2_S can directly form persulfides *via* reaction with oxidized cysteine residues (disulfides and sulfenic acids) as well with as the Cys-S-Zn sites of ZFs (Cuevasanta et al., 2015; Lange et al., 2019). Protein-persulfides can also be formed co-translationally with cysteinyl-tRNA synthetases (CARS) which functions cooperatively with the transsulfuration pathway, particularly during oxidative stress (Akaike et al., 2017; Sawa et al., 2020). Persulfidation impacts protein structure and can enhance or inhibit activity depending on the specific Cys residue that is modified. As such, the ‘signaling’ property of H_2_S can be described as modulating regulators and their function. ZFs are directly and indirectly affected by protein persulfidation. Directly, protein stability and activity are altered in the emerging examples of ZF persulfidation (Saha et al., 2016; Vandiver et al., 2013). ZFs are also indirectly induced by persulfidation of other proteins, such as p65 and MEK1, which both contribute to downstream upregulation of PARP-1 and other NFκB-associated ZFs (Dai et al., 2019; Sen et al., 2012; Zhao et al., 2014a).

## 5 ZF persulfidation and functional implications

### 5.1 ZF-persulfide studies to date

Our laboratory previously reported that the persulfidation of the tandem CCCH ZF domains of TTP leads to disruption of the protein’s structure and obviates TTP/RNA-binding (Lange et al., 2019). TTP-2D reacts with H_2_S when Zn(II) is bound to the protein and O_2_ is present. Cryo-electrospray-ionization Mass Spectrometry identified the persulfidated ZF. This method along with orthogonal techniques led to the identification of sulfur- and oxygen-based radicals formed during the persulfidation reaction. A mechanism whereby Zn(II) acts as a conduit for electron transfer between H_2_S and O_2_, activating O_2_ to form superoxide and other reactive species, leading to eventual disulfide formation and Zn(II) ejection was proposed. Cell experiments showed that MEF cells harvested from both WT and ΔCSE mice have similar basal levels of TTP; CSE contributes the majority of protein persulfidation in cells (Filipovic et al., 2018; Paul and Snyder, 2015). Thus, absence of persulfidation did not greatly affect TTP protein stability/abundance, as has been shown in some other examples of ZF-persulfides (Kalous et al., 2021; Saha et al., 2016; Vandiver et al., 2013). TNFα mRNA levels were decreased in the CSE knockout cells, connecting *in vitro* findings that persulfidation of TTP restricts its RNA binding activity. This loss of structure and function due to TTP persulfidation appears to be a regulatory mechanism for its activity, with H_2_S serving to “signal” mRNA processing.

We subsequently reported that persulfidation of ZFs is a common PTM. Analysis of persulfide specific proteomics data in multiple mammalian cell lines led to the identification of a large number of persulfidated ZFs. These include ZFs with different ligand sets and regulatory functions (e.g., transcription, translation, ubiquitination, etc.) (Li et al., 2024; Stoltzfus et al., 2024). The finding that ZFs with various ligand sets are persulfidated led us to investigate whether the chemistry that drives persulfidation is affected by ligand set. Using the TTP ZF peptide scaffold as the starting point, a series of mutants that offer one, two, three or four cysteine ligands per ZF domain were prepared and their reactivity with H_2_S evaluated. In all cases, the TTP ZF peptides were persulfidated by H_2_S, as long as Zn(II) was bound and O_2_ was present. These findings suggest a common chemistry amongst ZFs for persulfidation.

**FIGURE 8:**
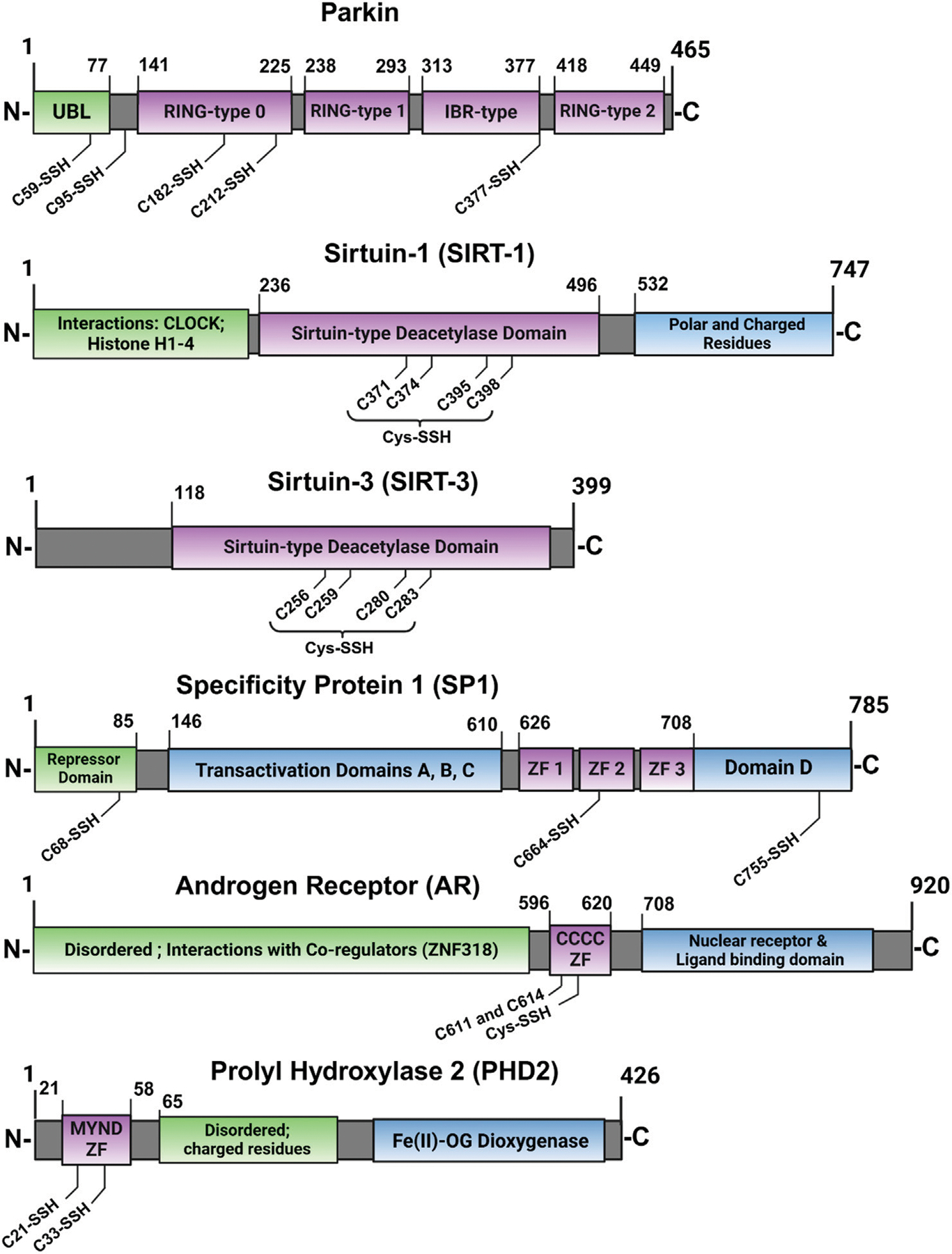
Cartoon depiction of ZFs for which persulfidation was identified for specific residues. Table 2 compiles this list of studied ZF-SSHs along with their Uniprot accession IDs and their percentage of Cys content based on their sequences (purple = ZF domain).

In addition to these proteomics data, there are scattered reports of isolated ZF-persulfides, shown in Figure 8, the earliest of which is Parkin (Vandiver et al., 2013). Parkin contains multiple RING ZF domains which confer E3 ubiquitin ligase activity. This study demonstrated functional activation of Parkin by persulfidation. Parkin was found to be physiologically persulfidated in healthy tissues but depleted in Parkinson’s disease (PD) patients and mice models, indicating the loss of Parkin persulfidation and activity as a pathogenic mechanism for sporadic Parkinson’s disease. A slow-releasing H_2_S-donor (GYY4137) improved Parkin-mediated ubiquitination and degradation of target protein, AIMP2, improving cell viability. As Parkin contains four ZF domains of various classes, this early work highlights the nuance of ZF persulfidation and the need for site-specific determination to consider the full protein sequence and the functional implications of persulfidation. The authors determined the specific residues of Parkin which are persulfidated using mammalian transfection, mutations of Parkin Cys residues, and mass spectrometry. Five Cys residues were determined to be persulfidated but modification of the catalytic C678, involved in ubiquitin transfer, was not observed, suggesting an allosteric effect on Parkin activity by persulfidation of the RING-type 0 (C182 and C212) and IBR-type (C377) ZF domains (Figure 8).

The classical ZF, SP1 (CCHH domain) has also been reported to be persulfidated. SPI acts as a transcriptional activator or repressor of GC-rich gene promoters (Beishline and Azizkhan-Clifford, 2015; Kaczynski et al., 2003; Vizcaino et al., 2015; Yin and Wang, 2021). SP1 was found to be upregulated during NFκB pathway stimulation by TNFα, leading to increased H_2_S production and persulfidation through SP1-mediated transcriptional activation of CSE (Sen et al., 2012). More recently, Saha and coworkers showed that SP1 is itself persulfidated, dependent on functional CBS, leading to enhanced transcription of VEGF-1 by SP1 (Saha et al., 2016). The authors again used an MS/MS technique to show that persulfidation of SP1 at C68 and C755 are necessary for optimal transcriptional activity in the maintenance of endothelial cell function. Although the persulfidated residues are outside of the ZF domains of SP1, they play a role in stabilizing the protein, resulting in higher SP1 protein levels, leading to optimal binding of its transcriptional elements. As CBS converts homocysteine to H_2_S in the transsulfuration pathway, knockdown of CBS led to hyperhomocysteinemia and a reduction in H_2_S and GSH levels (Miles and Kraus, 2004). Furthermore, SP1 had decreased binding to the VEGFR-2 promoter and endothelial cells displayed a phenotype with compromised chemotaxis. All outcomes were ameliorated by treatment with H_2_S, but not glutathione, demonstrating the importance of SP1 persulfidation mediated by proper CBS activity.

The MYND-type ZF (CCCC-CCHC) prolyl hydroxylase domain-containing protein 2 (PHD2) constitutively hydroxylates HIF-1α leading to ubiquitination and degradation (Dey et al., 2020; Maxwell et al., 1999). Hypoxic conditions lead to inhibition of PHD2 activity, stabilization of HIF-1α, complexation with HIF-1beta, and transcription of hypoxia-related survival genes. Dey and coworkers discovered that PHD2 is persulfidated at C21 and C33 residues of its MYND-type ZF domain (Figure 8), leading to enhanced hydroxylation of HIF-1α by PHD2 (Dey et al., 2020). Disruption of CBS in zebrafish lead to abnormal development, diminished persulfidation of PHD2, and stabilized HIF-1α. All phenotypes were rescued by H_2_S supplementation. These data support a role for persulfidation in healthy endothelial cell development.

The sirtuin family of ZFs has also been shown to be persulfidated (Kalous et al., 2021). This ZF family features a singular, conserved CCCC-type ZF domain that provides a structural element to deacetylate histones and activate transcription of stress-response genes. The SIRT1 and SIRT3 proteins have been shown to undergo persulfidation during various types of cell stress. Persulfidation improves SIRT1 protein levels in the cell by increasing Zn-binding affinity and reducing ubiquitination, both of which lead to enhanced protein stability and deacetylase activity (Dong et al., 2023; Du et al., 2019; Li et al., 2020; Wu et al., 2023). Similar observations have been made for SIRT3, localized in the mitochondria (Liu F. et al., 2023; Liu et al., 2020; Xiong et al., 2023; Yuan et al., 2019). Liu et al. established that persulfidation is essential for optimal SIRT3 activity in two ways: first, by the direct stabilization and activation of SIRT3 by persulfidation, and second by the indirect upregulation of SIRT3 transcription by Nrf-2, which is also indirectly activated by persulfidation of Keap1 (Liu et al., 2020). This Nrf-2/Keap1/SIRT3 persulfidation axis is perturbed during conditions of oxidative stress and can be rescued by exogenous NaHS supplementation. For both SIRT1 and SIRT3, the cumulative evidence highlights a role for ZF persulfidation leading to enhanced deacetylation activity, which is an essential factor for increased antioxidant gene expression by the sirtuin ZF family.

In contrast to the ZF-persulfides discussed so far, which have enhanced protein activity, there are also examples of protein inactivation by persulfidation, such as TTP-2D and androgen receptor (AR). Zhao and coworkers focused on AR, a CCCC type ZF that functions as a transcriptional activator of androgen-responsive gene elements, in androgen-resistance and the development of prostate cancer (Zhao et al., 2014b). They found that CSE expression was diminished in human prostate cancer tissue and androgen-resistant cell lines, suggesting a possible deficiency in H_2_S-associated signaling in this disease state. CSE overexpression repressed the expression of AR-target genes and remediated pathogenic phenotypes in mice models. They also found that mutation of the ZF domains of AR at C611 and C614 abrogated the rescue potential of CSE overexpression, indicating that persulfidation of these residues is required for regulation of AR *via* inhibition of AR dimerization and gene transcription.

### 5.2 Structure/function implications of ZF-SSH case studies

The broader biological prevalence and significance of ZF persulfidation have not been fully appreciated and reviewed. To connect the signaling role of H_2_S and persulfidation of Cys-rich ZFs, it is necessary to consider the local environment and location of specific residues found to be persulfidated. Of the six ZF-SSHs listed in Section 5.1 which were studied in cell (Parkin, SP1, PHD2, SIRT1, SIRT3, and AR), five ZFs had enhanced activity with persulfidation, as well as ameliorative effects in cells and tissue. What’s left to unravel are the connections between modified Cys residues and the fate of overall protein activity, although the commonality is activation by persulfidation. This may prove helpful in screening and characterizing potentially pathogenic mutations which lead to some loss-of-function due to diminished protein persulfidation.

One example is Parkin which was found to be persulfidated but to a diminished degree in Parkinson’s patients. Only three of five Cys mutants could be expressed and functionally assessed by iteratively mutating these residues from Cys to Ser. The three mutants (C59, C95, and C182) that were assessed are integral to the auto-regulatory inhibition of Parkin in its inactive, basal state. This suggests that persulfidation of these residues may contribute to the “opening” and activation of Parkin, as seen in the well-studied phosphorylation of Parkin by PINK1 (Barazzuol et al., 2020). Persulfidation of these residues likely does not directly enhance Parkin’s catalytic activity (as catalytic C678 was not modified), but rather it contributes to a greater pool of active Parkin and indirectly increases the ubiquitation of substrates.

As described in Section 5.1, SP1 persulfidation at C68 and C755 enhances its binding and transcriptional activation of VEGF (Saha et al., 2016). Separately, persulfidation of the second ZF domain at C664 is required for SP1-mediated inhibition of Krüppel-like factor 5 (KLF5) expression (Meng G. et al., 2016). These two cases illustrate that further characterization and partner/target binding studies are required for novel ZF-SSHs. Persulfidation of certain residues might enhance interactions with either a corepressing or a coactivating protein, depending on factors of the local environment such as surface accessibility. Further in-depth studies of ZF-SSHs can utilize the approaches from these examples and expand our basis of knowledge concerning the signals of persulfidation through ZFs.

### 5.3 Looking forward

A powerful tool in cell and animal models are persulfide-specific proteomic approaches which can determine enriched signaling pathways and new ZF-SSHs of interest. There are several persulfide-labeling approaches for proteomic identification (Li et al., 2024) and many can be used to corroborate protein-persulfides with non-reducing gel electrophoretic methods of labeled cell lysates. These labeling methods may be applied to other methods of visualization, such as microscopy and flow cytometry, to inform on cellular localization (Zivanovic et al., 2019).

As presented by the case studies mentioned here, experiments which identify residue-specific Cys persulfidation (i.e., mass spectrometry and/or protein mutation) are essential for ZF-SSHs. Furthermore, mapping these modifications to reported protein structures can aid in understanding the structural and functional consequences of ZF persulfidation. In cell, applying the techniques cited in Section 5.1 to newly identified ZF-SSHs will be helpful in uncovering the broader implications of ZF persulfidation and H_2_S signaling. Identified ZF-SSHs can be tested for site-specific persulfidation, which requires mammalian cell transfection, overexpression, and Cys-SSH identification by MS techniques. The cell systems are then utilized to iteratively mutate identified Cys residues and determine the functional fidelity of mutants (ex: Parkin-C182S). Additionally, knockout mice models can broadly (or specifically) reduce persulfidation and provide information about which H_2_S-generating enzymes contribute to the ZF-SSH of interest. By knocking out or inhibiting any of CSE, CBS, 3-MST, or CARS, direct connections between an enzyme and a ZF’s persulfidation can be elucidated. Similarly, biochemical studies on isolated ZF proteins and peptides, to further characterize persulfidation sites and determine the effects of persulfidation on protein activity will further broaden our understanding of ZF protein persulfidation and the role of H_2_S.

## 6 Potential therapies for chronic NFκB-associated syndromes

Dysfunction of NFκB signaling and ZFs is associated with metabolic syndromes, inflammation, cancer, and age-related diseases (Beishline and Azizkhan-Clifford, 2015; Bu et al., 2021; Hosea et al., 2023). Dysregulation of the transsulfuration pathway and protein persulfidation is likewise associated with these chronic conditions (Filipovic et al., 2018; Kabil and Banerjee, 2014). Antibody therapies targeting TNF and these associated pathologies are a large proportion of FDA-approved biologic therapies and are often conjugated with synthetic compounds, such as cytotoxins for cancer combination therapies, for multimodal effects (Lewis Phillips et al., 2008; Tiwari et al., 2016). *In lieu* of reviewing the intricacy of engineered antibodies, we refer readers to some excellent reviews (Leone et al., 2023; Mitoma et al., 2018; Morita et al., 2022; Shepard et al., 2017). In addition to the NFκB pathologies, ZFs and H_2_S are known to contribute to proper cardiovascular function (Meng et al., 2015; Pan et al., 2014; Pikkarainen et al., 2004); more recent examples, such as the SIRT ZFs (see Section 5) demonstrate the necessity of ZFs as conduits for persulfidation and H_2_S signaling. Much of the work to date connect persulfidation with perturbed NFκB signaling and excessive cytokine levels, suggesting that NFκB pathway is a molecular throughfare for ZF and H_2_S regulation. As more isolated ZF persulfides are characterized, it will become clearer as to how H_2_S-associated physiological effects are intertwined with ZF function/dysfunction.

Therapeutic efforts related to ZFs and sulfur homeostasis have been studied in several contexts, including gene editing therapies using artificial ZF domains and sulfur donating molecules which seek to restore imbalanced cellular small molecules.

### 6.1 ZF nucleases

While ZFs are now understood as ubiquitous regulators of transcription and translation, interest in gene editing precedes their discovery (Helene and Toulme, 1990; Kawasaki et al., 1996). ZFs have been studied for their potential in gene editing therapy due to their innate function of oligonucleotide binding. This specificity of binding is tunable for different targets (Bibikova et al., 2001; Kawasaki et al., 1996). ZF nucleases (ZFNs) are chimeric constructs containing multiple ZF domains for recognition of target genes, where each domain recognizes a triplet of nucleotide bases, fused with a nuclease domain (Porteus and Baltimore, 2003). The small recognition patterns provide ZFNs an advantage over other endonucleases, as the fingers can be modularly designed to increase sequence specificity (Hauschild-Quintern et al., 2013). ZFNs have been implemented in human and other mammalian cells, but translation to animal models and human clinical trials is still developing (Jabalameli et al., 2015).

The first human clinical trial using ZFNs was conducted in 2022 to treat mucopolysaccharidosis (n=12) and hemophilia B (n=1) (Harmatz et al., 2022). The authors found adequate safety and tolerance at all tested doses with evidence for successful gene editing and enhanced enzyme expression in liver tissues. However, the hemophilia B subject could not be assessed for gene-editing, and no long-term enzyme expression was observed in any patients. RNA-binding CCCH ZFs, such as MCPIP, are also in development as a potential ZFN design strategy for chronically elevated cytokines which may be disrupted by the stability and translational efficiency of their mRNA (Gaj et al., 2012; Garg et al., 2021; Liu et al., 2021; Paschon et al., 2019). Ultimately, the long-term efficacy of ZFN treatment, as seen in the clinical trial, needs improvement.

### 6.2 Sulfur donors

Glutathione and N-acetyl cysteine (NAC) have been popularized as antioxidant supplements in sports medicine and elsewhere, presumably acting by replenishing a depleted sulfur pool (Fernández-Lázaro et al., 2023). Dick and coworkers recently showed that the beneficial effects of NAC are due in part to endogenous H_2_S production from the provided Cys (Ezerina et al., 2018). As a result of increased H_2_S concentrations, persulfide species, including GSSH, were increased. Sulfane sulfur and persulfides have garnered increased interest over the last decade due to the ability of the former to store sulfur and generate various forms of protein- and small molecule-persulfides, which are associated with a multitude of positive biological outcomes in cell and animal studies (Dai et al., 2019; Doka et al., 2016; Donnarumma et al., 2017; Sun et al., 2020; Yu et al., 2023; Zhang et al., 2019; Zivanovic et al., 2019). The highly reactive nature of a persulfide makes it a better radical scavenger than other cellular thiols but also presents an obstacle to therapy as it may react before reaching its target (Filipovic et al., 2018; Fukuto et al., 2020). To overcome this, efforts have been made to create persulfide donor prodrugs which activate upon reaction with specific stimuli, such as superoxide, hydrogen peroxide, and photons (Bora et al., 2018; Chaudhuri et al., 2019; Wang et al., 2020). Other groups are dissecting the chemical reactivity of persulfides based on their local chemical environment, adding to both our fundamental understandings of protein persulfides and the tunable stability of persulfide-related therapies (Fosnacht et al., 2024). These therapeutic methods may restore ZF-SSH function and more broadly aid in biomarker discovery of diseases characterized by chronic inflammation.

## 7 Conclusion

Zn(II) and ZFs play a major role in the NFκB pathway, which responds to various stimuli for development, immunity, pathogen defense, cell death, etc. (van Loo and Bertrand, 2023). Although the types of ZF families, ligands, and functional partners are broad, all ZFs share the characteristic of binding Zn(II) in a tetrahedral coordination geometry to form a folded domain which is then functional. We are now learning these folded ZF domains can be modified *via* chemical transformations that affect protein function and offer a new layer of regulation. The transformation of cysteine into cysteine-persulfide, mediated by the gasotransmitter H_2_S, imparts numerous advantages for the fidelity of ZFs. On the molecular level, protein-persulfides have greater metal affinity and nucleophilicity than their thiol counterparts (Lau and Pluth, 2019). Furthermore, they are reversibly oxidized during stress and can be recovered by cellular reductants. In a broader context, persulfidation and H_2_S are ameliorative in models of chronic inflammation, such as cardiovascular, neurodegenerative, and immune diseases (Liu M. H. et al., 2023; Salti et al., 2024). ZFs regulate pathways related to these diseases and represent a sizeable superfamily of protein domains that are suitable messengers for H_2_S-related signaling. Persulfide specific proteomics data are uncovering multiple ZFs that are persulfidated. These findings open the door for experiments to decipher how persulfidation affects both specific ZFs and ZF rich signalling pathways. The understanding gained in these areas has the potential to inform on the development of new therapies for diseases impacted by aberrant sulfur homeostasis and ZF function.

## Supplementary Material

Table 1

Table 2

## Figures and Tables

**TABLE 1:** Listing of Uniprot-annotated Zinc Finger proteins (ZFs) which are involved in NF**κ**B signaling. ZFs above the bold line were identified in the KEGG analysis in Section 2.1; those below the bold line are included for their roles in NF**κ**B signaling and are discussed in this review.

Uniprot ID	Name	Gene Name	NFκB Effect
Q06187	BTK	Bruton tyrosine kinase (BTK)	Pro-inflammatory
Q9NYJ8	TAB2	TGF-beta activated kinase 1 (MAP3K7) binding protein 2 (TAB2)	Pro-inflammatory
Q8N5C8	TAB3	TGF-beta activated kinase 1 (MAP3K7) binding protein 3 (TAB3)	Pro-inflammatory
P21580	A20	TNF α induced protein 3 (TNFAIP3)	Anti-inflammatory
Q12933	TRAF2	TNF receptor associated factor 2 (TRAF2)	Pro-inflammatory
Q13114	TRAF3	TNF receptor associated factor 3 (TRAF3)	Anti-inflammatory
O00463	TRAF5	TNF receptor associated factor 5 (TRAF5)	Pro-inflammatory
Q9Y4K3	TRAF6	TNF receptor associated factor 6 (TRAF6)	Pro-inflammatory
Q9Y6K9	NEMO	Inhibitor of NFκB kinase regulatory subunit gamma (IκBκG)	Pro-inflammatory
P09874	PARP1	Poly (ADP-ribose) polymerase 1 (PARP1)	Anti-inflammatory
Q8N2W9	PIAS4	Protein inhibitor of activated STAT 4 (PIAS4)	Pro-inflammatory
P98170	XIAP	X-linked inhibitor of apoptosis (XIAP)	Pro-inflammatory
Q13490	cIAP1	Baculoviral IAP repeat containing 2 (BIRC2)	Pro-inflammatory
Q13489	cIAP2	Baculoviral IAP repeat containing 3 (BIRC3)	Pro-inflammatory
P05771	PKC-β	Protein kinase C beta (PRKCB)	Pro-inflammatory
Q04759	PKC-θ	Protein kinase C theta (PRKCQ)	Pro-inflammatory
Q14258	TRIM25	Tripartite motif containing 25 (TRIM25)	Anti-inflammatory
P26651	TTP	Tristetraprolin (TTP); mRNA decay activator protein ZFP36	Anti-inflammatory
Q5D1E8	ZC12A	Monocyte chemotactic protein-induced protein 1 (MCPIP1)	Anti-inflammatory
Q5TC82	RC3H1	Roquin; RING finger and C3H zinc finger protein 1	Anti-inflammatory
Q9H0F6	SHRPN	SHARPIN; Shank-associated RH domain-interacting protein	Pro-inflammatory
Q9BYM8	HOIL-1	Heme-oxidized IRP2 ubiquitin ligase 1 (HOIL-1)	Pro-inflammatory
Q96EP0	RNF31	HOIL-1interacting protein (HOIP); RING finger protein 31	Pro-inflammatory
Q96CV9	OPTN	Optineurin; FIP2; Huntington-interacting protein 7 (HIP-7)	Anti-inflammatory
Q8TE49	OTU7A	OTU domain-containing protein 7A; ZFP Cezanne 2	Anti-inflammatory
Q92844	TANK	TRAF-interacting protein (TANK)	Anti-inflammatory

**TABLE 2:** ZF-persulfides (ZF-SSHs) examples from literature and their Cys abundance as a function of their full sequence and the sequence excerpt from the beginning of ZF 1 to the end of any additional ZFs in the given protein.

Uniprot ID	Protein names	Totalsequence length (residues)	% Cys abundance		ZF-only length	ZF-only residue range	Refs.
			Total sequence	ZF domains only			
P08047	Transcription factor Sp1	785	1.4	7.3	82	626–708	Saha et al. (2016), Meng et al. (2016)
O60260	E3 ubiquitin-protein ligase parkin (Parkin)	465	7.5	10.1	308	141–449	Vandiver et al. (2013)
P10275	Androgen receptor (AR)	920	2.9	16.7	60	560–620	Zhao et al. (2014b)
Q96EB6	NAD-dependent protein deacetylase sirtuin-1 (SIRT1)	747	2.5	4.2	260	236–496	Dong et al. (2023), Du et al. (2019), Li et al. (2020), Wu et al. (2023)
Q9NTG7	NAD-dependent protein deacetylase sirtuin-3, mitochondrial (SIRT3)	399	1.8	1.9	262	118–380	Liu et al. (2023a), Liu et al. (2020), Xiong et al. (2023), Yuan et al. (2019)
Q9GZT9	Prolyl hydroxylase domain-containing protein 2 (PHD2)	426	3.5	18.9	37	21–58	Dey et al. (2020)
P26651	mRNA decay activator protein ZFP36 (Tristetraprolin, TTP)	326	3.1	9.1	66	103–169	Lange et al. (2019)
